# Mapping and estimating the total living biomass and carbon in low-biomass woodlands using Landsat 8 CDR data

**DOI:** 10.1186/s13021-016-0055-8

**Published:** 2016-06-24

**Authors:** Belachew Gizachew, Svein Solberg, Erik Næsset, Terje Gobakken, Ole Martin Bollandsås, Johannes Breidenbach, Eliakimu Zahabu, Ernest William Mauya

**Affiliations:** 1 Norwegian Institute of Bioeconomy Research, Post Box 115, 1431 Ås, Norway; 2 Department of Natural Resource Management, Norwegian University of Life Sciences, Post Box 5003, 1432 Ås, Norway; 3 Faculty of Forestry and Nature Conservation, Sokoine University of Agriculture, P.O. Box 3009, Chuo Kikuu, Morogoro, Tanzania

**Keywords:** Biomass, Carbon, Modeling, Miombo woodlands, REDD+, NDVI

## Abstract

**Background:**

A functional forest carbon measuring, reporting and verification (MRV) system to support climate change mitigation policies, such as REDD+, requires estimates of forest biomass carbon, as an input to estimate emissions. A combination of field inventory and remote sensing is expected to provide those data. By linking Landsat 8 and forest inventory data, we (1) developed linear mixed effects models for total living biomass (TLB) estimation as a function of spectral variables, (2) developed a 30 m resolution map of the total living carbon (TLC), and (3) estimated the total TLB stock of the study area. Inventory data consisted of tree measurements from 500 plots in 63 clusters in a 15,700 km^2^ study area, in miombo woodlands of Tanzania. The Landsat 8 data comprised two climate data record images covering the inventory area.

**Results:**

We found a linear relationship between TLB and Landsat 8 derived spectral variables, and there was no clear evidence of spectral data saturation at higher biomass values. The root-mean-square error of the values predicted by the linear model linking the TLB and the normalized difference vegetation index (NDVI) is equal to 44 t/ha (49 % of the mean value). The estimated TLB for the study area was 140 Mt, with a mean TLB density of 81 t/ha, and a 95 % confidence interval of 74–88 t/ha. We mapped the distribution of TLC of the study area using the TLB model, where TLC was estimated at 47 % of TLB.

**Conclusion:**

The low biomass in the miombo woodlands, and the absence of a spectral data saturation problem suggested that Landsat 8 derived NDVI is suitable auxiliary information for carbon monitoring in the context of REDD+, for low-biomass, open-canopy woodlands.

## Background

Deforestation and forest degradation in the tropics continue to be a significant source of global carbon dioxide (CO_2_) emissions and the largest source of CO_2_ in the developing world [1]. After years of negotiations, the United Nations Framework Convention for Climate Change (UNFCCC) gradually recognized a mitigation mechanism for reducing emissions from deforestation and forest degradation and forest conservation, sustainable management of forests and enhancing forest carbon stock in the tropics and subtropics (REDD+) [2]. REDD+ deploys results-based finance to incentivize emissions reduction, based on a functional forest carbon measuring, reporting and verification (MRV) system [3]. Nevertheless, technical challenges in MRV [4, 5] have, thus far, substantially contributed to the lack of progress for implementation of REDD+ on the ground.

A functional MRV to support REDD+ requires estimates of the area of forest loss and gain and the corresponding carbon stock and changes [3, 6]. These data are needed for the estimation of the actual emissions and the construction of forest reference emissions level (FREL), a benchmark against which the actual emissions are compared [7]. A combination of field inventory and remote sensing is expected to provide those data. National forest inventories (NFIs) with repeated measurements are often lacking for most of the forests and woodlands of Africa. On the other hand, there exists a wide range of remote sensing technologies, including sensors on-board aircraft and space-based platforms. Landsat is one of the most attractive remote sensors because its images are freely available and have medium resolution, large spatial and frequent temporal coverage [8].

Landsat data have been widely used in forest aboveground biomass (AGB) estimation, commonly through developing empirical relationships between AGB or other forest characteristics and spectral indices such as the normalized difference vegetation index (NDVI) derived from satellite data [e.g., 8–17]. Large scale and coarse resolution biomass or carbon mapping using satellite imagery for tropical forests in Africa has been demonstrated [10, 18–20]. Fine to medium scale carbon maps offer more detailed and locally accurate auxiliary information in the context of REDD+. For instance, the majority of the REDD+ countries that have submitted a FREL proposal to the UNFCCC [21] used medium resolution maps based, largely, on Landsat to extract activity data, i.e., forest area change. Biome or forest type specific biomass models or carbon maps with higher spatial resolution can also provide estimates of carbon densities [22], that can be used as emissions factors that are more reliable than for instance the IPCC default values [23].

For Landsat based approaches, cloud and cloud shadows [24] as well as canopy reflectance data-saturation has been reported as a major problem for high biomass, closed canopy forests and high leaf area index [25]; particularly for the tropical rainforests [24] and secondary successional forests [26]. Another problem of Landsat based approaches is related to the impact of shadows caused by canopy in complex stand structures and relatively complex topography [27, 28]. Furthermore, interference by understory vegetation and soil in open woodlands may require correction when spectral indices such as NDVI are used [29]. Yet, in savannah-type open Mediterranean evergreen woodlands, models with spectral indices including NDVI had comparable or better predictive capabilities of tree canopy cover [30]. Tropical forests, also, consist of extensive open canopy and low biomass woodlands, such as the miombo woodlands. The miombo woodlands are the dominant forest types in many African countries. In Tanzania, the woodlands dominated by miombo make up to 90 % of the forest area [31]. The miombo woodlands are of major significance in Tanzania, as a source of livelihood, in supporting biodiversity and maintaining the local hydrological cycle [32, 33]. Moreover, with the advent of REDD+, the miombo woodlands provided Tanzania with a potential to receive financial benefits through the carbon they store. These woodlands, however, are under continuous pressure from land clearing for agriculture and charcoal production as well as fire [33]. Reliable methods for biomass estimation and carbon mapping are needed to monitor the dynamics and to support activities and decisions in the context of REDD+.

The Landsat 8 sensor has come with design improvements over Landsat 7 ETM+, including narrower near-infrared wavebands, higher signal-to-noise ratio, and greater radiometric sensitivity [34]. It has also shown a better capability in land cover mapping and is expected to present new opportunities for understanding the contribution of forest ecosystems to the carbon cycle [35]. The current study aims at testing the potential of Landsat 8 for estimation of total living biomass (TLB), i.e., the sum of the above and belowground woody biomass in miombo woodlands. The specific objectives are to (1) develop empirical models to estimate TLB from spectral indices derived from Landsat 8 data, and (2) develop a 30 m resolution spatial map of total living carbon (TLC) by applying the TLB model, and (3) estimate the TLB and TLC stock and density where, the TLC was estimated at 47 % of the TLB [36].

## Methods

### Study area

**Fig. 1:**
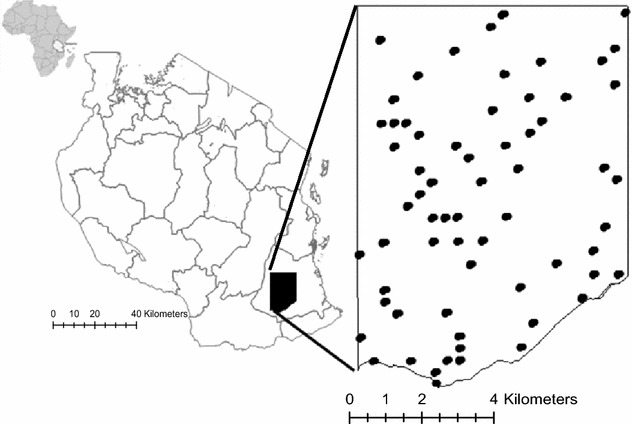
Location of the study area and the clusters, from *left* to *right*: Tanzania in Africa, study area in Lindi region of Tanzania (*polygon* in *dark*), and the distribution of sample clusters (*dark dots*) in the study area polygon

The study area is a polygon of 15,700 km^2^, originally delineated as part of a previous project [37], located in Liwale district, southeastern Tanzania (36°50′–38°48′E; and 8°–10°50′S (Fig. 1). The miombo woodlands are the dominant vegetation type in the region with more than 100 tree species including timber species of *Brachystegia* sp., *Julbernadia* sp. and *Pterocarpus angolensis*, with trees up to 35 m height [38, 39]. The mean annual temperature is 25 °C, ranging from 20–30 °C. Annual rainfall ranges from 600 to 900 mm with a bi-modal pattern. The long rainy season occurs from March to May, while a dry season occurs from July to October, followed by a short rainy season from November to January.

Human activities are common in the district such as shifting cultivation and harvesting for charcoal and fuel wood, and land clearing for agriculture. Forest fire is also an important factor in the miombo ecosystem. Up to 50 % of the miombo woodland area is affected by predominantly small-sized fires with less than 2 years of fire return intervals, which prevent the occurrence of large intense fires [40]. The northern part of the study area falls within the territory of the Selous game reserve, one of the largest fauna reserves of the world.

### Sampling design and field inventory data

Field inventory data came from 500 plots grouped in 63 clusters. The field plots were originally established in 2011, as part of the National Forest Resources Monitoring and Assessment (NAFORMA), which is the national forest inventory of Tanzania [31]. The NAFORMA plots were established using a systematic double sampling for stratification design with clustered plots [31]. The first-phase comprised L-shaped clusters containing 6–10 plots each. The clusters were located at the nodes of a 5 × 5 km grid across mainland Tanzania. Each cluster was assigned to one of 18 predefined strata, based on predicted growing stock volume, time consumption for cluster measurements and the slope of the terrain [31]. Within each stratum, second-phase samples of clusters were selected systematically among the first-phase clusters using optimal allocation [41].Higher sampling intensities were achieved for strata with higher growing stock volume, which implies that spending relatively larger resources (more plots) on high-volume strata was an effective means to reduce the overall uncertainty of growing stock volume estimates. Only those clusters selected during the second phase of sampling were measured in the field. The distance between plots within a cluster was 250 m, while the distance between clusters varied from 5 to 45 km. Details of the NAFORMA sampling design and the inventory procedures, and illustrations are presented in Tomppo et al. [31].

The established plots were revisited and measured in 2012 [37], and then again in 2014. Although most of the field plots fall in miombo woodlands, plots in agricultural fields and other vegetation types were also included in the inventory data. In the current study, we used the data from the 2014 field visit. The field measurements in 2014 were conducted in the period January–July and followed a similar procedure with that of NAFORMA in 2011. Differential Global Navigation Satellite Systems were used to determine the coordinates of the center point of each plot. Two Topcon Legacy 40-channels dual frequency receivers observing both pseudo-range and carrier phase of the Global Positioning System (GPS), along with the Global Navigation Satellite System (GLONASS) were used as rover (on the plot) and base station respectively. The estimated accuracy of the planimetric plot coordinates ranged from 0.01 to 1.41 m, with an average of 0.13 m. Each plot of size 707 m^2^ consisted of four concentric circles of radius 2, 5, 10 and 15 m. In each circle, all trees of diameter at breast height (dbh) greater than or equal to 1, 5, 10, and 20 cm, respectively, were measured for dbh. Every fifth tree on a plot was selected as a sample tree for height measurement. For trees without height measurements, total heights were estimated using local height-dbh models of the form in Eq. 1 [cf. 42] developed using the sample trees.

1 $$h = 1.3 + b_{0} (dbh)^{b1}$$

where *h* = total height, b_0_ and b_1_ are coefficients estimated from the measured sample trees.

AGB and the belowground biomass (BGB) of each sampled tree in the plots were estimated using the allometric biomass models (Eqs. 2, 3) developed for miombo woodlands in Tanzania [43],

2 $$AGB = 0.0763 (dbh)^{2.2046} h^{0.4918}$$

3 $$BGB = 0.1766 (dbh)^{1.7844} h^{0.3434}$$

where *dbh* (cm) and *h* (m) are defined above and *AGB* and *BGB* are in kg.

AGB and BGB predictions of each tree were scaled up to a per hectare level based on plot size information given by dbh-thresholds of the concentric circles. The total living biomass (TLB) is the sum of AGB and BGB and the total living carbon (TLC) was estimated at 47 % of TLB [36]. Table 1 presents plot summary statstics for the variables of interest.

**Table 1:** Summary statistics of aboveground biomass (AGB), belowground biomass (BGB), total living biomass (TLB) and total living carbon (TLC)

Variable (t/ha)	Minimum	Mean	Std. Dev.	Maximum
TLB	0	88.49	64.52	436.20
AGB	0	62.10	48.73	350.30
BGB	0	26.39	16.88	89.97
TLC	0	41.59	30.33	205.00

Values are based on plot measurements (n = 500). The minimum (0) are from plots without trees

### Landsat 8 data and spectral variables for modeling biomass

Two Landsat 8 surface reflectance climate data record (CDR) images, with 30 m resolution, (Worldwide Reference System 2: path 166 and row 66 and 67) were acquired through the USGS Earth Explorer (earthexplorer.usgs.gov). Both images were from 31 July 2014, and were almost cloud free over the study area. The data acquisition date corresponded to the cool early dry season [44], which is expected to offer a good contrast between understory vegetation and the woody biomass for open woodland, as the understory vegetation begins to dry off. The two images were pre-processed by the provider, i.e., corrected for atmospheric conditions and topography, and with cloud and cloud shadow masking. The CDR data were derived from Landsat 8 Operational Land Imager (OLI) using Landsat Ecosystem Disturbance Adaptive Processing System (LEDAPS) software [45], which applies moderate resolution imaging spectroradiometer (MODIS) atmospheric correction routines to Level-1 scenes [46].

For each NAFORMA plot, we extracted seven spectral reflectance values (bands 1–7) from the pixel containing the plot center, using the default extraction procedure in ArcGIS The surface reflectance specification and the center wavelengths of each Landsat 8 band are available in [46]. We also tested a second approach which extracts the weighted mean value of the four nearest pixels, weighting the contribution of each pixel by its fraction of the 707 m^2^ plot area. The second approach was thought to reduce sampling error in the case of spatial mis-registration of the image. However, a paired t test for the differences between spectral indices developed based on the values extracted using the two extraction methods did not show any significant difference (*p* > 0.05). Furthermore, the spectral variables from the two methods produced similar correlation coefficients with biomass, with no gain in using the second approach which was finally discarded.

Seven spectral indices (Table 2) were calculated using the different spectral band combinations. These particular indices were selected not only because they are related to land surface features and vegetation biomass, but also because they have some advantages for practical applications since they are readily provided from the USGS on demand [46].

**Table 2:** Spectral indices derived from the spectral band reflectance values

Index	Equations (spectral bands)	References
Normalized difference vegetation index (NDVI)	NDVI = (NIR − red)/(NIR + red)	[47]
Enhanced vegetation index	EVI = (NIR − red)/(NIR + 6 (red) − 7.5 (blue) + 1)	[48]
Soil adjusted vegetation index (SAVI)	SAVI = ((NIR − red)/(NIR + red + 0.5)) (1.5)	[49]
Modified soil adjusted vegetation index (MSAVI)	MSAVI = (2 (NIR) + 1 − sqrt ((2 (NIR) + 1)^2^ − 8 (NIR − red)))/2	[50, 51]
Normalized difference moisture index (NDMI)	NDMI = (NIR − SWIR1)/(NIR + SWIR1)	[52]
Normalized burn ratio (NBR)	NBR = (NIR − SWIR2)/(NIR + SWIR2)	[53]
Normalized burn ratio-2 (NBR2)	NBR2 = (SWIR1 − SWIR2)/(SWIR1 + SWIR2)	[53]

*NIR* near infra-red (band 5), *SWIR1* short wave infra-red (band 6), *SWIR2* short wave infra-red 2 (band 7), *sqrt* square root

### Model development

In order to examine the relationship between TLB and the 14 spectral variables (seven spectral bands and seven spectral indices in Table 2), we calculated Pearson’s correlation coefficient (ρ), and also visually analyzed the scatter plots of TLB against those spectral variables. All the 14 spectral variables correlated significantly with TLB, but they were also highly inter-correlated (*p* < 0.005). The latter would lead to a multicollinearity problem, if those variables were used simultaneously as predictor variables in a regression model. We, therefore, evaluated the strength of the relationship between TLB and each spectral variable using correlation and preliminary regression analysis to select the best explanatory variable for model development.

As a means to understand the form of the relationship between TLB and the spectral variables, we aggregated the plots in the respective clusters and graphically evaluated the scatter plots of TLB against the spectral values. While the aggregated cluster level data were used for graphical analysis, data from the individual plots (n = 500) were used for the final model development.

The scatter plots of the TLB against the spectral values suggested linear models may sufficiently describe the relationships. Due to the hierarchical structure of the data (plots nested in clusters) a linear mixed modeling approach was appropriate [cf., 54, 55]. We evaluated alternative mixed models with the random intercept, random slopes or both, on the basis of improvements in Akaike’s Information Criterion (AIC) and the Bayesian Information Criterion (BIC). The mixed models with random intercept and slope (that varies with cluster) (Eq. 4) produced the smallest AIC and BIC.

4 $$Y_{ij } = (\alpha + a_{j} ) + (\beta + b_{j} )\left( X \right)_{ij} + e_{ij} ,$$

where *Y* denotes the dependent variables (TLB), *X* denotes the independent spectral variables and *i* indexes plots within clusters (1, 2,…, 8), and *j* indexes clusters (1, 2,…,63). Coefficients *α* and *β* represent the intercept and the slope of the fixed part of the model, respectively, and *a*_*j*_ and *b*_*j*_ are the random intercept and slope, respectively (deviations around the value *α* and *β*). The *a*_*j*_ and *b*_*j*_ are assumed to be distributed normally with mean zero, and G-variance–covariance matrix given as:

$$\left( {\begin{array}{*{20}c} {a_{j} } \\ {b_{j} } \\ \end{array} } \right) \, \sim \,N\,\left[ {\left( {\begin{array}{*{20}c} 0 \\ 0 \\ \end{array} } \right),G = \left[ {\begin{array}{*{20}c} {\sigma_{int}^{2} } & {\sigma_{int,slope}^{2} } \\ {\sigma_{int,slope}^{2} } & {\sigma_{slope}^{2} } \\ \end{array} } \right]} \right]$$

In the G variance–covariance matrix, the $\sigma_{int}^{2}$ and $\sigma_{slope}^{2}$ represent variance components for the random intercept and slope, respectively; the ($\sigma_{int,\,slope}^{2}$) are covariance components representing the correlation between the random intercept and the random slope.

The *e*_*ij*_ are the residuals of *i*th plot in the *j*th cluster and are assumed to be normally distributed with mean 0 and variance σ^2^, i.e., *e*_*ij*_ ~ N (0, σ^2^). We fitted the model using the Restricted Maximum Likelihood (REML) with unstructured variance–covariance, using the mixed procedure in SAS [55]. We applied the empirical option for the mixed effects procedure in SAS, also known as the “sandwich” estimator which computes a robust variance–covariance matrix of the fixed effects parameters using an asymptotically consistent estimator [56–59].

We evaluated model performance based on root mean square error (RMSE) and RMSE as a percent of the mean of the field inventory TLB (%RMSE), and absolute bias (Eqs. 5, 6, 7) and residual diagnostics. To evaluate the accuracy of the final TLB model, we used a leave-one-out cross validation (LOOCV) in which we left out a cluster at a time.

5 $$RMSE = \sqrt {\frac{1}{n}\mathop \sum \limits_{i = 1}^{n} \left( { y_{i} - \hat{y}_{i} } \right)^{2} }$$

6 $$\% RMSE = \frac{RMSE}{{\bar{y}_{i} }}*100$$

7 $$Absolute\,bias = \frac{1}{n}\mathop \sum \limits_{i = 1}^{n} (y_{i} - \hat{y}_{i} ) ,$$

where *n* is number of plots, ${\hat{\text{y}}}_{i}$ is predicted TLB for the *i*th plot using model (4) fitted without the plots in the same cluster as the *i*th plot, and (y_*i*_) is the observed TLB for the *i*th plot.

### Mapping and estimation of total living carbon

We applied the best TLB model to the NDVI composite of the two Landsat 8 CDR images and estimated the mean TLB density and the TLB stock of the entire study area. To estimate a 95 % confidence interval of the mean TLB density and total biomass stock, we used a model-based variance estimator for the population (the study area) as described in McRoberts [60], i.e.,

8 $$\widehat{Var} \left( {\hat{\bar{Y}}} \right) = \,\bar{X}^{T} \hat{\varvec{\varSigma }}\bar{X} ,$$

where, $\widehat{Var} \left( {\hat{\bar{Y}}} \right)$ is the variance estimator of the population mean estimate $\hat{\bar{Y}}$. $\hat{\bar{Y}}$ was estimated as the mean of the pixel-wise model predictions across the entire study area, $\bar{X}$ is the vector for the population mean of the explanatory variables including the intercept, *T* stands for transpose of a matrix, and $\hat{\varvec{\varSigma }}$ is the variance–covariance matrix of the fixed parameter estimates (intercept and slope of the model, Eq. 4). We compared the TLB density estimate of the study area with a value estimated from three recent pan-tropical biomass maps of Baccini et al. [20], Saatchi et al. [18] and Avitabile et al. [61]. The later was developed by combining the data sets of the earlier two [18, 20]. Since these biomass maps are for AGB, the mean ratio of BGB to AGB of 0.43 from our field data (n = 500) was used to calculate the corresponding TLB.

We applied the TLB model to the two NDVI composite images covering the study area to construct a 30 m resolution TLC map, where TLC is estimated at 47 % of TLB. To further demonstrate the utility of the model for local carbon mapping and stock estimation, we extracted the carbon map of 13 wards, using the ward boundaries as a mask. We compared the estimated TLC values with values based on field data from previous works in the region. In the absence of other maps for that particular year and season, we visually compared extracts of the carbon distribution pattern from our map with image extracts from Google Earth [62] of the same year and season. The 13 wards have a combined area of 4780 km^2^ and included Liwale town, the major town in Liwale district. We selected this area, particularly because it consisted of settlements as well as a number of human activities, such as land clearing for agriculture, fuel wood and charcoal, as well as fire, which are likely to influence the forest cover and thus carbon stock and its distribution.

## Results

### Relationships and regression models for TLB

**Fig. 2:**
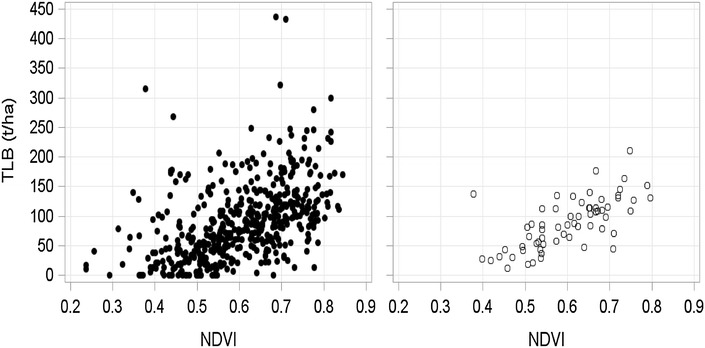
*Scatter plots* of the total living biomass (TLB) (t/ha) against the NDVI [plot values (*left*), n = 500, and cluster mean values (*right*), n = 63]

All the 14 spectral variables were significantly correlated to TLB and inter-correlated amongst each other (*p* < 0.005). The Pearson correlation (ρ) between the spectral variables and TLB ranged from |0.18| to |0.50| (Table 3). The indices NDVI, EVI, SAVI, and MSAVI and the spectral bands-Blue and Red correlated most strongly with TLB (ρ ≥ |0.45|). It appeared, therefore, that any one of these variables, particularly the EVI, would be a useful predictor in the model. Nevertheless, the correlation (ρ) of EVI, although slightly better, was not convincingly larger (Table 3) to forgo NDVI, which is popular, intuitive and easier to interpret and to compare with other works. NDVI was thus selected as a single predictor for the final model. The large variability in the preliminary scatter plots of TLB against spectral data of individual plots limited our ability to detect absence or presence of a data saturation effect. It is likely that the small size of the field plots contributed to the observed large variability. However, the scatter plots of the NDVI against TLB, particularly those based on mean values at cluster level data showed no sign of saturation at higher biomass (Fig. 2) and suggested that the observed relationships were linear.

**Table 3:** Pearson correlation coefficients between spectral variables and TLB (t/ha) of plots (n = 500)

Landsat 8 spectral bands						
Coastal	Blue	Green	Red	NIR	SWIR1	SWIR2
−0.43	−0.45	−0.44	−0.48	0.18	−0.38	−0.43

Spectral vegetation indices						
NDVI	EVI	SAVI	MSAVI	NDMI	NBR1	NBR2
0.49	0.50	0.49	0.49	0.44	0.45	0.43

Prob > |ρ| under Ho: ρ = 0, and ρ is significant (*p* < 0.005*)* for all the spectral variables. The level of confidence α = 0.05

Parameter estimates and the associated statistics of the linear mixed model are presented in Table 4, along with the fit statistics. Plots of the residuals against the fitted TLB values, except for few data points of NDVI values less than 0.2, showed that the residuals had a constant variance over the range of TLB. The estimated covariance matrix used for the variance estimators of the mean and the total TLB for the entire study is given in Table 5.

**Table 4:** Parameter estimates of the fixed part of the TLB model (*p* < *0.001*) with NDVI as a predictor

	Intercept ($\alpha$)	Slope ($\beta$)	RMSE (t/ha)	%RMSE	Absolute bias (t/ha)
Model	−84.22	280.93	43.66	49.00	0.00
Standard error	24.15	40.20			
LOOCV			56.00	63.00	0.00

Statistics presented for the model construction and cross validation (LOOCV)

**Table 5:** Empirical covariance matrix for the fixed effect parameters ($\hat{\varvec{\varSigma }}$) where values in the diagonal are the variances of the intercept and the slope, respectively, and the off diagonal elements are the covariance between the two

	α	β
α	583.14	−960.27
β	−960.27	1615.77

### Biomass and carbon mapping and estimation

**Fig. 3:**
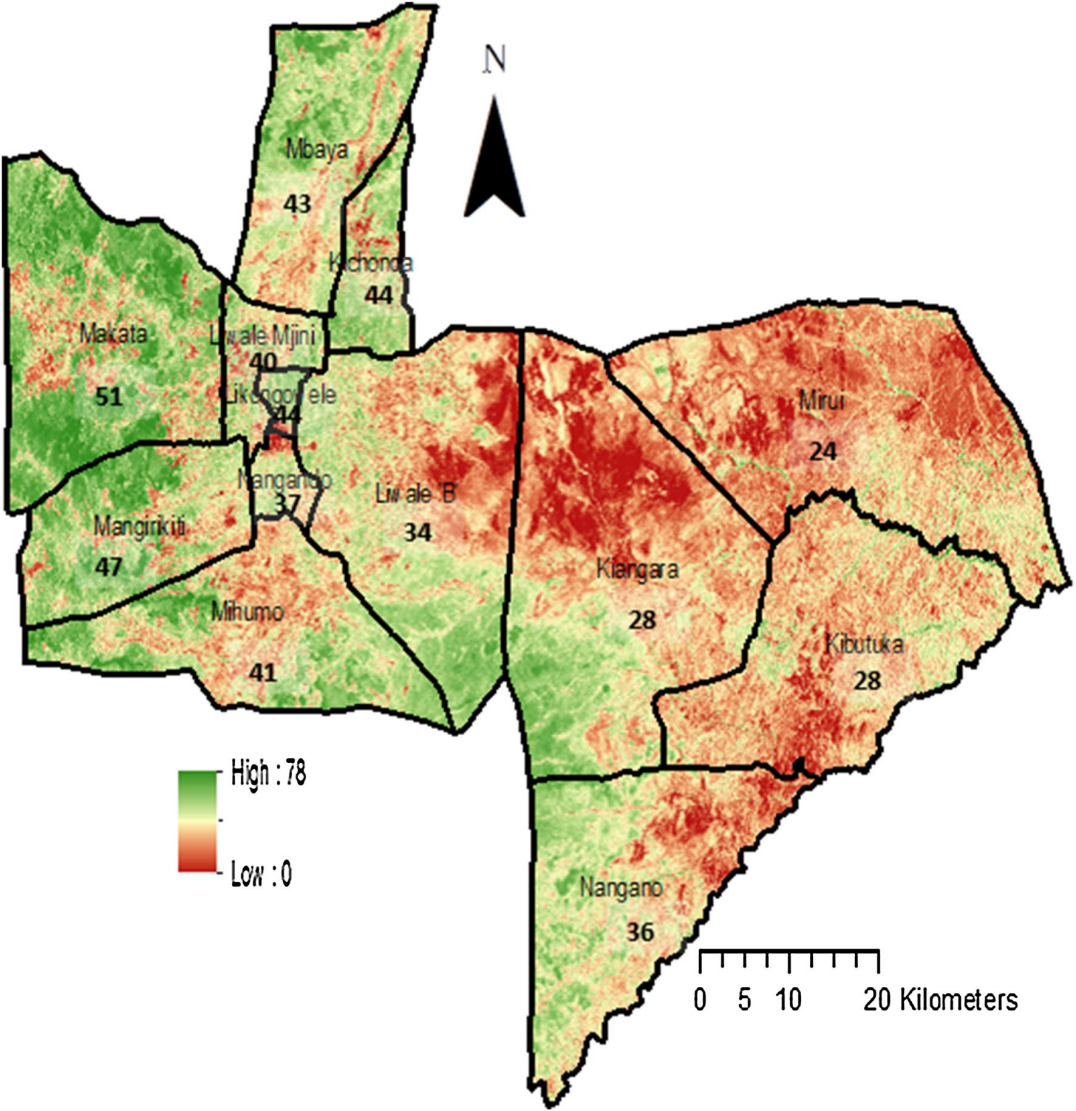
Total living carbon (TLC) density map of 13 wards in Liwale district. *Numbers* in each ward are mean TLC (t/ha). The reference, Liwale town falls within the boundaries of four wards, Nangando, Likongowele, Liwale Mjini, and Liwale B

**Fig. 4:**
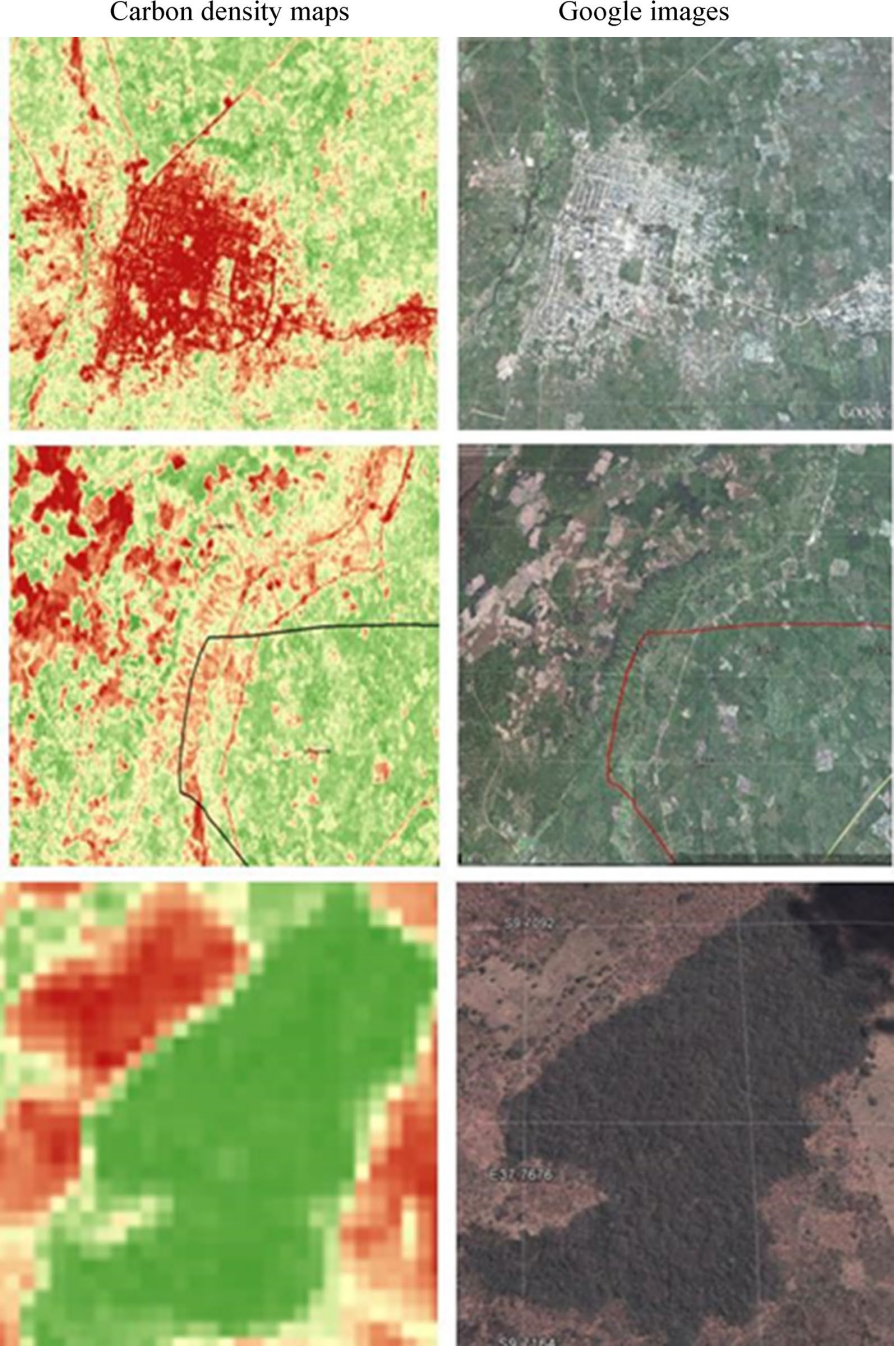
Visual comparison between the extracts of the 30 m resolution total living carbon (TLC) map in Liwale district *left panels* (based on this study)—and extracts of Google Earth images—*right panels*, showing settlement areas (Liwale town)—*top two panels*, clear cut areas in Liwale Mjini ward—*middle panels*, and a dense forest in Makata ward—*bottom panels*. The TLC density map and google earth image extracts are from the year 2014. In the extracts of the map (*left panels*), *color* ranges are from low (*deep red*) to high (*green*) TLC values

The estimated mean TLB density for the study area was 81 t/ha with a 95 % confidence interval of ±7 t/ha. The total TLB stock was estimated at 140 Mt, with a 95 % confidence interval of ±14 Mt. We used the TLC map, developed based on the TLB model, to estimate carbon stock by ward and visualize the carbon distribution pattern among 13 wards for the year 2014 (Fig. 3). The mean TLC varied considerably among the 13 wards, ranging from 24 to 51 t/ha. For example, the four most eastern wards in the map had carbon densities of 25–29 t/ha, which was less than the average of the entire study area. In contrast, the four most western wards had carbon densities of 42–51 t/ha, which was higher than the average for the study area. The spatial distribution of predicted TLC corresponded well to the extracts from Google Earth images (Fig. 4). As can be expected, settlement areas and farmlands had low predicted TLC, and the dark green areas representing forests depict higher values.

## Discussion

### Relationship between biomass and NDVI

The linear relationship between the TLB and NDVI suggested that spectral data saturation is not a problem when using Landsat for biomass and carbon monitoring in miombo woodlands. Data saturation related to optical sensors is reported as a major limitation or source of uncertainty in forest biomass estimation in complex stand structures and closed canopy forests [10, 22, 24, 63]. A similar study in miombo woodlands in Mozambique [64] found a significant linear correlation (ρ = 0.30) between NDVI and the AGB. In dry land open canopy vegetation in Argentina as well, NDVI data saturation was, not observed [65]. Pixel-level NDVI is influenced by the degree of absorption of the red wavelengths by chlorophyll, which is proportional to the amount of chlorophyll in the leaf and the reflectance of near infrared (NIR) radiation, which is proportional to green leaf density [47]. The relatively low aboveground biomass in the open canopy woodlands might have resulted in the low reflectance values in the NIR band, which overcomes the saturation problem of the NDVI. The NIR, although statistically significant, produced the weakest correlation with TLB (ρ = 0.18), suggesting less green leaf density and thus low reflectance in open canopy woodlands. For boreal forests with evergreen conifers and relatively closed canopy forests in Canada [66, 67] and mountain birch forests in Finland [68], NIR was considered a strong predictor of stand attributes such as height, crown closure or AGB.

Geographic factors, such as elevation, slope and aspect as well as shadows are also known to affect spectral reflectance values, and thus compromise biomass estimation performances of models based on spectral variables [24]. However, NDVI, as a ratio of two bands, is expected to correct potential errors due to topography and shade and compensate the variation in illumination resulting from terrain features [28]. Lyon et al. [27] compared seven vegetation indices to detect land-cover change in a Chiapas, Mexico and reported that the NDVI was least affected by local geographic factors such as slope and aspect.

The absence of data saturation facilitated development of a linear model between TLB and NDVI. Model fit statistics, i.e., RMSE (%) and less bias in the cross validation (Table 4), along with the constant residual errors (not shown) suggested the model has a comparable accuracy with previous works based on similar approach. These results are comparable to many other models for vegetation properties based on Landsat data, where model %RMSE values were in the range of 21–50 % [13, 28, 67, 69]. Other studies that have reported higher model accuracies [e.g., 68, 70] were based on data from single species stands or plantations. Furthermore, many of these studies attempt to model AGB, rather than TLB, mostly because of the relative ease of estimating AGB or availability of allometric models for AGB only. The model presented here enables direct estimation of TLB, including the BGB. In the absence of BGB data, the default ratio of 0.28 between the BGB and AGB [36] is recommended for tropical dry forests for estimating TLB. This default ratio is much lower than the mean BGB to AGB ratio of 0.43 in our data. The BGB, although significantly related to the AGB, in this study was independently calculated for each plot based or the below ground tree allometric equations. The TLB model can, therefore, be considered an important contribution for miombo woodlands, where data availability for the BGB remains a challenge.

The performance of the model showed that NDVI is a good predictor of TLB in open woodlands. Yet, there remains a sizeable amount of unexplained variation in the data, which can be attributed to a number of sources. Miombo woodlands are subject to distinct seasonal processes, and the beginning of the dry season marks a change in phenological cycles and leaf density might decrease, compared to the rainy season when ecosystem productivity is higher. NDVI, as a greenness indicator, cannot clearly distinguish variations in biomass related to phenology, the complexity of forest species and canopy structure. Shrub and grass layers were not measured in the field for the BGB and AGB calculations, but their contribution to the total biomass may be small. Nevertheless, in the spectral variable extraction, the effects of the shrub and grass layer on canopy reflectance are unknown, but could be large, depending on the intensity of greenness or the grass layer phenology during image acquisition. Other sources of uncertainty include a potential mismatch between the field coordinates and the points of spectral variable extraction. The field plots are circular with a size of 707 m^2^ while the spectral variables are extracted from square pixels of size 900 m^2^. Furthermore, tree level BGB and AGB were derived from allometric models with sizeable errors that are likely to propagate through to plots, pixels and thus to the TLB model [e.g. 71]. Therefore, model sensitivity to those factors, including phenology, stand structure, and their application in areas of low biomass need to be investigated. Incorporating auxiliary biophysical predictors, such as land-use, land-cover or vegetation structure information may improve model accuracy, by reducing the spectral variability within those biophysical predictors [e.g. see 10, 67, 72].

Other remote sensing data sources with higher resolution may produce more accurate models and estimates of better precision. In a recent study, conducted in parts of our study area, Næsset et al. [73] estimated the relative efficiency (RE), i.e., variance of the field-based estimate relative to the variance of that of the different remotely sensed data to quantify contribution of different remote sensing data sources to improve precision of AGB estimates. Their results showed that RE values were 3.6 for ALS, 3.3 for RapidEye, and 2.8 for InSAR; while for Landsat and PALSAR the RE values were only 1.3–1.4. The latter two contributed only marginally to improve precision. The limited spatial and temporal coverage as well as the costs of acquiring and processing those types of data, however, limit their extensive applicability to larger areas. In contrast, the use of Landsat 8 data for modeling purposes can be justified as the data cover large areas and are freely available. Furthermore, Landsat 8 came with considerable improvements in sensor signal-to-noise performance and associated improvements in radiometric resolution [35]. Dube and Mutanga [70] reported an improved accuracy for estimation of AGB, for instance for *E. dunii* plantations, by using Landsat 8 (RMSE = 26.54 % of the mean) compared to Landsat 7 ETM+ (RMSE = 35.30 % of the mean). Furthermore, the availability of higher order data (Landsat 8 CDR) products, for free, reduces the efforts and costs of data preprocessing, which otherwise require complex procedures.

### Carbon mapping and estimation

The carbon mapping and estimation was based on the TLB model and estimation. The mean TLB density of 81 t/ha for the study area is higher than the estimate based on Avitabile et al. [61], 48 t/ha, and lower than that of Baccini et al. [20], 138 t/ha; but closer to that of Saatchi et al. [18], 73 t/ha. Since these tropical biomass maps and our maps were based on different data acquisition systems and methods, calibrated with different field data sets and have different spatial accuracies, the large differences among them in the biomass estimates for smaller localities are not unexpected. Previous studies showed that maps of Baccini [20] and Saatchi [18] often show very strong local differences, but at national and global scales their estimates tend to converge [61, 74], mostly due to compensation of contrasting estimates when averaging over large areas [61]. Moreover, data for these biomass maps were acquired on average around 2007 [61], while that of our study are from 2014. The dynamics in the miombo woodlands such as clearing and fire as well as reforestation means that, maps based on data from the same year or season are needed for a more confident comparison.

The developed medium spatial resolution (30 m) map for prediction of TLC, along with the model-based variance estimates for the predicted values of the TLC, demonstrated the potential utility of Landsat 8 for forest carbon monitoring. One of the major applications of carbon maps includes use of the map as auxiliary data in estimation of carbon density and stocks, and the corresponding land area. Forest carbon accounting principles in the context of REDD+ draws on the basic formula where the emissions from REDD+ activities are calculated as estimates of areas (activity data) multiplied by estimates of change in carbon density in those areas (emissions factors) [36]. Both activity data and emissions factors are required to be as accurate as practicable [36], since the total uncertainty of emissions is calculated by combining the uncertainties of the two.

The lack of ecosystem specific knowledge on carbon stock (the basis for emissions factors), and methods to determine the activity data (areas) are considered the major limitations in the progress for REDD+, particularly in Africa [4, 5]. The approach presented here can help determining local carbon density and stock for a defined area, in combination with available land use or land cover maps as a mask. But for a given reporting region or sub-region such as REDD+ project areas, carbon maps based on Landsat 8 calibrated with field observations can be used to estimate carbon density and total carbon stock within the boundary, so as to maximize potential financial benefits from payments or credits for carbon.

The estimated mean carbon density of 38 t/ha for the entire study area is very close to the IPCC Tier 1 default value of 36 t/ha for tropical dry forests in Africa [23]. But the wide range of TLC (25–51 t/ha) among the 13 wards indicates a large spatial variation and suggests the utility of spatial maps to visualize the carbon distributions and estimate carbon density or stock for smaller administrative units or localities where carbon reporting might be required. The estimated mean and range for the 13 wards are close to the living carbon for miombo woodlands in Mozambique, (average 29.8 t/ha ± 13.07) [75] and 28–36 t/ha [76]. Tanzanian studies report only the AGB estimates. In an attempt to compare with our results, we added the BGB to their AGB estimates, using the BGB to AGB ratio of 0.43 in our data. Our estimates of TLC density range correspond with the 39–54 t/ha estimated by Kashindye et al. [77] and 15–47 t/ha estimated by Shirima et al. [78]. While the two studies are from different areas of the miombo woodlands in Tanzania, another study from three villages located within the boundary of our study area presented a range of 24–28 t/ha [79], which is at the lower range of our estimate. It is, however, important to note that the other studies considered only plots with trees while our estimates came from all land use types, including agricultural lands.

The other potential application of the carbon map includes detection of spatial patterns of carbon density in connection with human induced disturbances such as settlements, fire or forest clearing. As expected, lower carbon densities are exhibited near and inside settlements such as Liwale town (Fig. 4, top panels) and in wards located east of Liwale town (Fig. 3). Furthermore, Google Earth images from June 2014 and our carbon map (Fig. 4, middle panels) show areas of low carbon corresponding to patches of clear cut areas, perhaps cleared for agriculture or wood extraction and roads. Clearing for agriculture was estimated to result in an annual forest loss of 10–25 ha in Liwale district [80], which would amount to 380–950 tons of carbon, given a carbon density estimate of 38 t/ha in this study. On the other hand, higher biomass vegetation can be shown for instance, in dense forests (Fig. 4, the bottom two panels). Fire is also an important component of the miombo woodland ecosystem, affecting up to 30 % of the woodlands in Liwale district [80]. The low carbon densities in eastern wards such as Kiangara and Mirui (Fig. 3) correspond with the presence of large forest fires in these areas during the year 2013 [62]. Furthermore, there was an indication of fire scar detected using visual analysis of the fire-sensitive short-wave infrared (band 7) complemented by the Near Infra-Red (band 5) and the visible (band 2) [cf., 81] of the same Landsat 8 data used for this study.

Our approach may also offer a potential for detection of carbon change due to deforestation, fire or land use change. This can be accomplished by image-differencing or calculating the differences in TLB values between two points in time. Nevertheless, several factors influence the reflectance values from different satellite images, including vegetation phenology and stand structure, atmospheric conditions when images were taken, and differences in sensors and image pre-processing procedures. This requires recalibration of the model coefficients to apply to other NDVI composite images of different areas and dates of acquisition. These factors are not addressed here, but understanding and identifying these sources of uncertainty will help to refine the model and improve its applicability to carbon mapping and estimation and applications to other seasons or regions.

The regression model slightly under predicts carbon for pixels with low NDVI, but will still be reliable for miombo woodlands, because biomes typically comprised of open woodlands and savannah exhibit NDVI values greater than 0.4 [82], and 95 % of the field plots in our study also exhibited and NDVI larger than 0.4. Although plots without trees and thus lower NDVI are few in the data, we had a true probability sample and the sampling frame covered other land use types including agriculture, and burnt areas which are common in the miombo woodlands.

## Conclusion

Landsat 8 CDR provides suitable data for monitoring of forest biomass and carbon in miombo woodlands. The open canopy and low biomass of the miombo woodlands means that there was low or no data saturation problem, which otherwise is a well-known challenge in using Landsat sensors. This property facilitated the development of a simple linear model which provides the basis for mapping forest carbon, estimating carbon stock and detecting its spatial distribution. The developed TLB model and carbon map also includes the below ground biomass, which otherwise are often estimated using a default root to shoot ratio from AGB. The Approaches presented here i.e., modeling, carbon mapping, and estimation can assist to estimate carbon and detect areas of low or high forest carbon, which are relevant to REDD+ activities, particularly, deforestation and enhancing carbon stock.

## Abbreviations

AGB: above-ground biomass
ALS: Airborne Laser Scanning
BGB: below-ground biomass
CDR: climate data record
InSAR: Interferometric Synthetic Aperture RADAR
IPCC: Intergovernmental Panel on Climate Change
MRV: measuring, reporting and verification
NDVI: normalized difference vegetation index
REDD+: reduce emissions from deforestation and forest degradation forest conservation, sustainable management of forests and enhancement of forest carbon stocks
RMSE: root mean square error
TLB: total living biomass
TLC: total living carbon
UNFCCC: United Nations Framework Convention on Climate Change
